# Multispectral Fluorescence Imaging as a Tool to Distinguish Pelvic Lymphatic Drainage Patterns During Robot-assisted Lymph Node Dissection in Prostate Cancer

**DOI:** 10.1245/s10434-024-16423-1

**Published:** 2024-11-19

**Authors:** Anne-Claire Berrens, Tessa Buckle, Matthias N. van Oosterom, Leon J. Slof, Pim J. van Leeuwen, Esther M. K. Wit, Hilda A. de Barros, Jakko A. Nieuwenhuijzen, Elise M. Bekers, Maarten L. Donswijk, Fijs W. B. van Leeuwen, Henk G. van der Poel

**Affiliations:** 1 Department of Urology, Netherlands Cancer Institute-Antoni van Leeuwenhoek Hospital, Amsterdam, The Netherlands; 2 Interventional Molecular Imaging Laboratory, Leiden University Medical Center, Leiden, The Netherlands; 3 Department of Urology, Amsterdam University Medical Center, VUmc, Amsterdam, The Netherlands; 4 Department of Pathology, Netherlands Cancer Institute-Antoni van Leeuwenhoek Hospital, Amsterdam, The Netherlands; 5 Department of Nuclear Medicine, Netherlands Cancer Institute-Antoni van Leeuwenhoek Hospital, Amsterdam, The Netherlands

**Keywords:** Fluorescence guided surgery, Image-guided surgery, Indocyanine green, Lymphedema, Lymph node dissection, Prostate cancer, Robot-assisted surgery, Sentinel node

## Abstract

**Background:**

The invasive nature of extended pelvic lymph node dissection (ePLND) prompts the need for alternative lymphatic mapping technologies. To change the focus to “sparing nodes that are not involved,” the first step is to research the feasibility of intraoperatively distinguishing the lymph drainage patterns of the prostate from healthy organs.

**Methods:**

We performed a prospective study (NCT05120973) that included 16 patients who underwent a robot-assisted radical prostatectomy + ePLND + sentinel node (using indocyanine green-^99m^Tc-nanocolloid). After general anesthesia, a second fluorescent dye (fluorescein) was injected unilaterally in two deposits into the intradermis of the upper leg (*n* = 8) or abdominal wall (*n* = 8), because these are the most common locations of lymphedema in prostate cancer surgery. To distinguish between the drainage patterns, in vivo and ex vivo multispectral fluorescence imaging was performed by using a fluorescence endoscope.

**Results:**

Indocyanine green and fluorescein were visible in the same regions within the ePLND-template and co-accumulated in lymph vessels in vivo. At histopathology, fluorescein was seen in only 10 of 370 lymph nodes (possibly owing to tracer properties), none of which overlapped with indocyanine green and none were tumor-positive. Administration of fluorescein did not result in discomfort or abnormal postoperative recovery.

**Conclusions:**

Multispectral imaging can be used to distinguish lymphatic drainage patterns. Our in vivo findings indicate that within the ePLND-template, lymphatic drainage patterns of the prostate at least partly overlap with those of upper leg and abdominal wall. The properties of fluorescein render it unsuitable for confirmation of fluorescence at histopathology.

**Supplementary Information:**

The online version contains supplementary material available at 10.1245/s10434-024-16423-1.

The European Association of Urology guidelines suggest consulting prostate cancer (PCa) patients about the advantages and disadvantages of extended pelvic lymph node dissection (ePLND) when nomogram- or classification-assessed risk of nodal involvement (LNI) exceeds 7%.^1–3^ Surgical disruption of lymphatic flow, crucial for tissue-fluid balance, can lead to complications.^4–6^ Studies have indicated that the risk of complications increases with the number of lymph nodes (LNs) dissected.^5,7^ Lymphoceles (up to 15%) and lymphedema (up to 14%) are reported most frequently.^8,9^ The invasive nature of ePLND prompts the need for alternative lymphatic mapping to prevent dissection of lymphatic anatomies unrelated to the prostate.

To facilitate a less invasive node dissection template, the distinction of drainage patterns from the prostate and healthy organs is necessary during surgery. Lymphangiographic or sentinel node (SN) targeting techniques are valuable.^10,11^ For lymphangiography, relatively small (<10 nm) organic dyes that flow freely through the lymphatics are used (e.g., blue dye, fluorescein, or indocyanine green [ICG]).^12,13^ These lymphatic tracers can be detected in white-light or through fluorescence imaging. Sentinel node procedures rely on the use of larger (>20 nm) colloidal particles taken up by macrophages residing in LNs (e.g., nanocolloid).^14,15^ In prostate cancer, on the morning of SN surgery a hybrid tracer combining ICG and Technetium (^99m^Tc) is administered, followed by static planar lymphoscintigraphy and single photon emission computed tomography (SPECT)/CT to guide intraoperative identification.^15,16^

Preclinical studies have indicated that multispectral fluorescence imaging was feasible to differentiate between lymphatic drainage patterns of different anatomies.^17^ Insights that recently resulted in multispectral fluorescence studies in porcine models, where the lymphatic drainage of the prostate (ICG-Nanocolloid; *λ*_em max_ = 820 nm) could be separated from that of the lower limbs (fluorescein; *λ*_em max_ = 515 nm) during robot-assisted surgery.^18^ In humans, this has not yet been confirmed but shows promise; multicolor/multispectral (fluorescence) imaging was proven effective to study the lymphatic drainage of only one organ, e.g., prostate or endometrium.^19,20^

The purpose of this translational trial was to objectify the technical feasibility of multispectral intraoperative fluorescence imaging to separate lymphatic drainage patterns of prostate (ICG-technetium(^99m^Tc)-nanocolloid;) versus the upper leg or abdominal wall (fluorescein) within the ePLND-template in humans during robot-assisted surgery.

## Methods

### Study Design and Patient Population

This single-arm, single-center prospective feasibility study was approved by the local ethics committee at the Netherlands Cancer Institute-Antoni van Leeuwenhoek Hospital (NCI-AVL), Amsterdam, The Netherlands (NCT05120973). All patients provided written informed consent.

Sixteen male patients aged 18 years or older, with histopathologically confirmed PCa and a calculated risk of LNI >5% according to Briganti (2012), who were scheduled to undergo robot-assisted radical prostatectomy with ePLND, were included between March 2022 and August 2023.^2^ Based on the EAU guidelines, patients routinely underwent PSMA PET/CT.^1^

### SN Identification

**Fig. 1:**
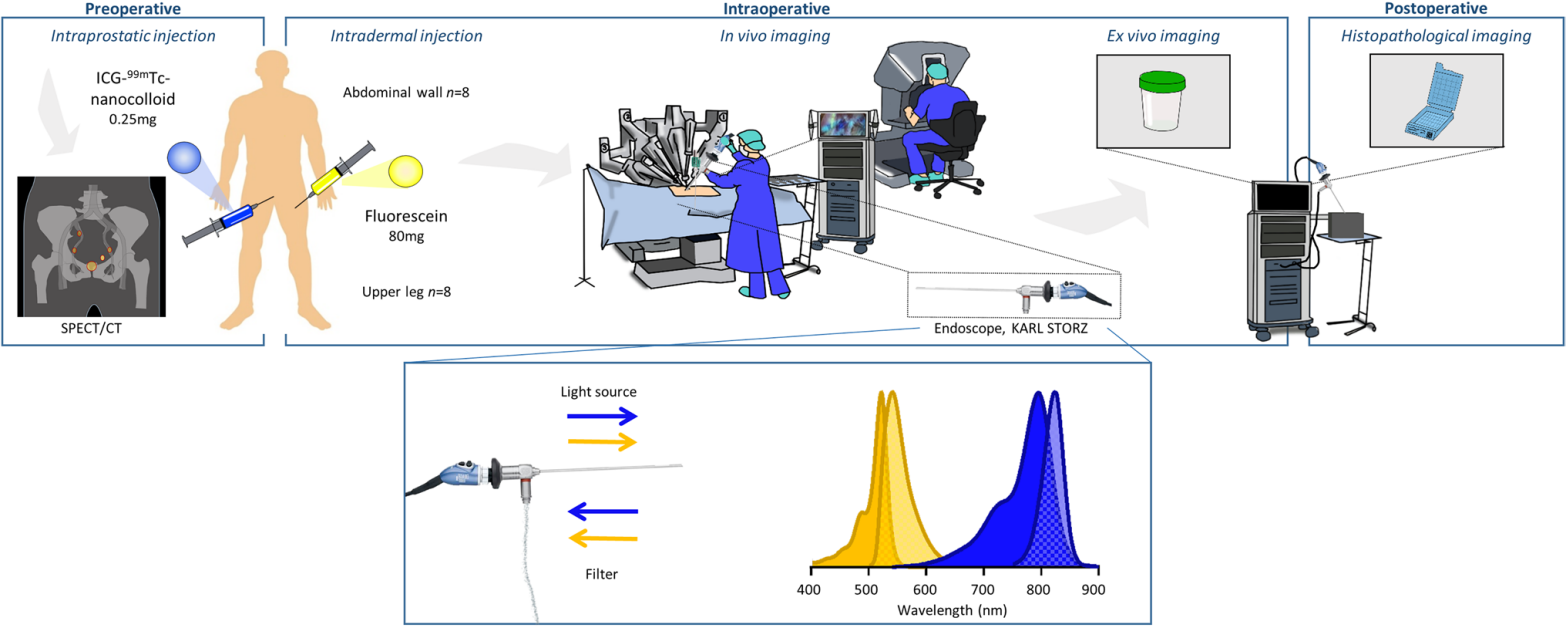
Illustration of perioperative study workflow. According to sentinel node procedure, ICG-^99m^Tc-nanocolloid was injected into the prostate approximately 5 h preoperatively. Fluorescein was injected intradermally after general anesthesia into the upper leg or abdominal wall, followed by in and ex vivo imaging directly after removal of tissue in the operating room. After 48 h, histopathological imaging was performed on formalin-fixed tissue

ICG-^99m^Tc-nanocolloid (Nanocoll^®^/Nanoscan^®^; GE Healthcare BV, Leiderdorp, the Netherlands) was administered 5 h prior to surgery in four deposits of 0.5 ml into the prostate.^16^ At 15 minutes and 2 h post tracer injection, lymphatic mapping was performed by using static planar lymphoscintigraphy and SPECT/CT (Fig. 1; Supplementary 1).

### Lymphangiography Upper Leg or Abdominal Wall

After administering general anesthesia, fluorescein (80 mg, *T*^1/2^ 24 min) was injected unilaterally into the dermis in two deposits of 2 ml each, either at the medial and lateral side of the upper leg (*n* = 8) or at the left or right lower quadrant of the abdominal wall (*n* = 8) (Fig. 1; Supplementary 1). The side of injection was chosen based on the side showing the most SNs on SPECT/CT to optimally investigate possible converging drainage patterns. The surgery was performed by four robotic surgeons from the Prostate Cancer Network Netherlands, specialized in prostatectomies and SN procedures. On both sides, SN and ePLND was performed. The included lymph nodes in the ePLND-template were removed as obturator combined with internal iliac nodes and external iliac nodes. Malignant looking non-SNs outside the template (Cloquet’s node/common iliac/presacral/etc.), and SNs from all regions were sent to pathology separately.

### In Vivo Fluorescence Imaging

After docking the robot (da Vinci Xi Surgical System, Intuitive Surgical, Inc, Sunnyvale, CA), the surgeon commenced with SN and ePLND on the side of the fluorescein injection. The integrated Firefly fluorescence camera of the robot was used for initial assessment. It is essential to recognize that Firefly is not able to differentiate between fluorescein or ICG.^21^ Based on van den Berg et al.,^19^ at standardized moments during the procedure, secondary fluorescence imaging was performed by using an endoscope combined with the Image 1 HUB HD + D-light P (ICG; *λ*_ex max_ = 800 nm, *λ*_em max_ = 820 nm) and D-light C (fluorescein; *λ*_ex max_ = 488 nm, *λ*_em max_ = 515 nm) light sources (KARL STORZ Endoscopie Nederland B.V., Amersfoort, the Netherlands), which allowed visualization of both ICG and fluorescein. The endoscope was maneuvered by the bedside assistant. The view of the endoscope could be observed by the operating surgeon by integrating the images using the Tile Pro input. Additional multispectral fluorescence imaging was employed in regions where Firefly indicated a fluorescent signal to distinguish ICG from fluorescein.

### Ex Vivo (Back Table) Evaluation

Ex vivo imaging was performed to validate in vivo findings immediately after removal. All excised tissue specimens were examined for the presence of ICG, fluorescein, a combination of both, or absence of staining, using the KARL STORZ endoscope (Fig. 1). Tissue samples were collected in separate containers or in large tissue samples, and areas of interest were marked with sutures. *Ex vivo* evaluated samples of four patients who had not received fluorescein served as negative control.

### Histopathological Evaluation

After *ex vivo* imaging, tissue samples were put in formalin according to clinical protocol. At least 48 h later, the samples were examined again for the presence of ICG or fluorescein by using the same endoscope and sent for histopathological examination with hematoxylin and eosin staining. From one patient and one control, formalin-fixed, paraffin-embedded tissue samples were analyzed for the presence of ICG and fluorescein to determine tracer signal after paraffin-embedding. Dedicated uropathologists microscopically evaluated all slides.

### Safety and Follow-Up

Exclusion criteria were prior abdominal or inguinal surgery, a history of severe allergic reaction, and known conditions of the kidney or thyroid. Because of the recognized increased risk of anaphylactic reactions to fluorescein, patients taking beta-blockers were excluded from participation.^22^ The injection of fluorescein into the skin was an off-label use. Consequently, the injection sites were monitored for redness or swelling at 15-min intervals throughout the procedure. Immediately after surgery and the next morning, the injection sites were assessed for presence of redness, swelling, and fluorescence using an ultraviolet (UV) flashlight. The use of ICG-^99m^Tc-nanocolloid in SN procedures in PCa is routine care at the NCI-AvL. All adverse events and postoperative surgical complications within 90 days were assessed according to the Clavien-Dindo classification.^23^

### Study Endpoints

The primary outcome was the technical feasibility of using multispectral fluorescence imaging to distinguish different lymphatic drainage patterns intraoperatively in primary PCa patients. For *in vivo* lymph staining in >10 patients, the method was considered feasible. Secondary outcomes included the determination whether and where the lymphatic drainage pattern of the upper leg/abdominal wall converges with that of the prostate and correlation of presence of ICG-^99m^Tc-nanocolloid and fluorescein in histopathological samples with tumor.

### Statistical Analysis

The sample size of 16 patients was not based on statistical power considerations owing to the exploratory nature of the study but on the basis of practical and clinical considerations. Interim analysis was performed after five patients. Descriptive statistics are presented as frequencies with percentages or medians and interquartile range (IQR). Statistical tests were done by using IBM SPSS version 29. A *p* value < 0.05 was considered statistically significant.

## Results

**Fig. 2:**
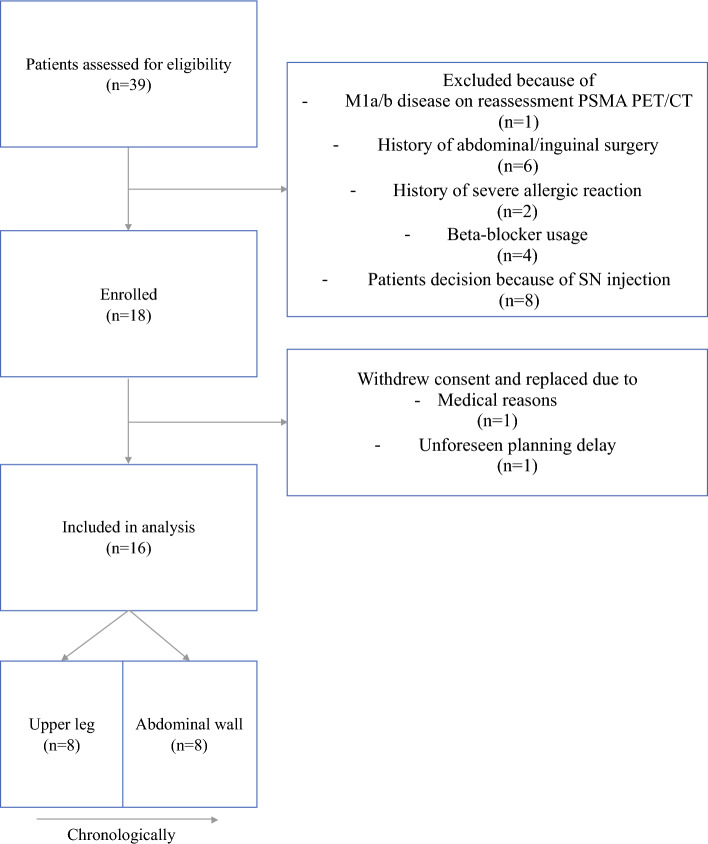
Patient assessment and enrollment

Eighteen patients were enrolled, of which 16 patients were included. Two patients withdrew consent before surgery. One because of medical reasons, and one because of an unforeseen planning delay (Fig. 2). Median age at surgery was 66.5 years (interquartile range [IQR] 61–69), median initial PSA was 15.3 ng/ml (IQR 7.8–21.9), and 8 of 16 (50%) of patients were overweight (body mass index > 25.0). All patients were miN0 on preoperative prostate-specific membrane antigen (PSMA) PET/CT with a median nomogram-assessed risk of LNI of 18.9% (IQR 12.5–33.7) (detailed description on patient demographics in Supplementary 2; race/ethnicity is not generally reported in patient files and therefore is not included in the table). SPECT/CT revealed a median of three visible SNs (IQR 3–4). Median operating room duration was 244 min (range 222–256.5).

### Intraoperative Imaging—Prostate (ICG) Versus Abdominal Wall or Upper Leg (Fluorescein)

**Fig. 3:**
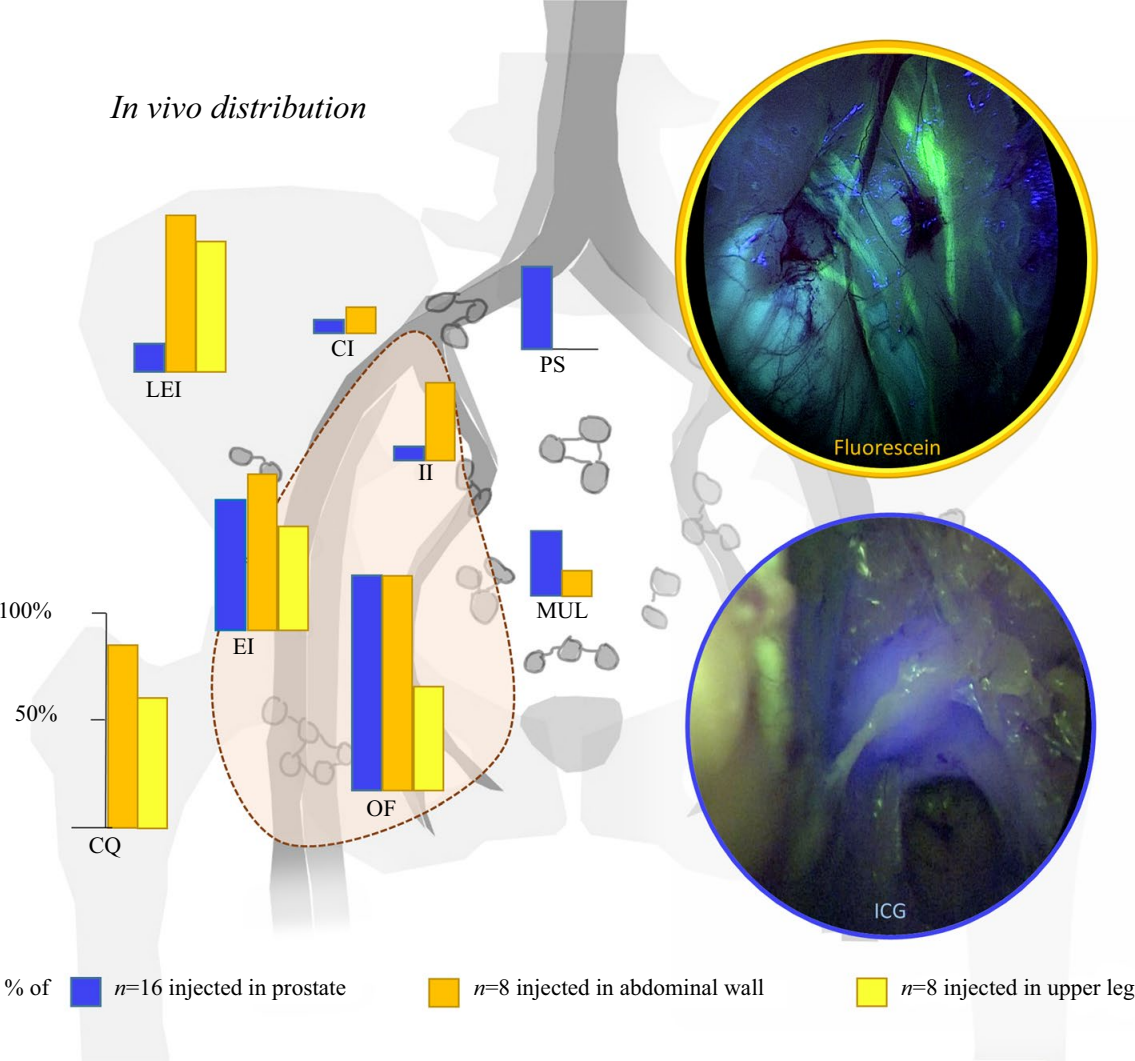
Unilateral representation of distribution of fluorescein (upper leg; yellow, and abdominal wall; orange) and ICG-^99m^Tc-nanocolloid (prostate; blue) with examples of in vivo KARL STORZ endoscope imaging filtered for fluorescein (yellow) and ICG (blue). *CQ* Cloquet’s; *OF* obturator fossa; *EI* external iliac; *LEI* lateral to external iliac; *II* internal iliac; *CI* common iliac; *PS* presacral/pararectal; *MUL* medial to umbilical ligament/paravesical

The median interval between injection and surgical imaging was 4.9 h (IQR 4.8–5.1) for ICG-^99m^Tc- nanocolloid and 16 min (IQR 15.0–21.5) for fluorescein. In vivo most of the fluorescein resided in lymph vessels (LV) (~48 LVs vs. 2 LNs), whereas ICG accumulated primarily in LNs (~42 SNs vs. ~3 LVs). Fluorescein was not observed at the contralateral side of the injection. Combining all unilateral visualizations, fluorescein was observed both within the ePLND-template (obturator fossa (75% of patients), external iliac (63%), internal iliac (19%)) and outside the template (lateral to external iliac artery 69%, common iliac (6%), and crossing the umbilical ligament (6%)) (Table 1; Fig. 3). Fluorescein was located in Cloquet’s node in 75%, whereas ICG was not seen at all at this location. ICG, following the SN pathway of the prostate, was also seen within the ePLND-template (obturator fossa (100%), external iliac (63%), internal iliac (13%)). Indocyanine green (or SNs) outside the ePLND-template was distributed lateral to external iliac (13%), around common iliac (6%), pararectal/presacral (25%), crossing the umbilical ligament/paravesical (19%) (Table 1; Fig. 3). Twice it was observed that fluorescein and ICG overlapped in a lymph vessel, both times in the internal iliac region.

**Table 1:** Surgical and histopathological outcomes

Parameter	Overall (*n* = 16)			Upper leg group (*n* = 8)		Abdominal wall group (*n* = 8)	
*Preoperative*							
Median injected dose ^99m^Tc, MBq (IQR)	219.6 (216.3–224.4)			220.1 (213.6–231.9)		219.3 (216.6–220.9)	
Median time injection ICG to first intraoperative imaging, hours (IQR)	4.9 (4.8–5.1)			4.9 (4.8–5.3)		4.9(4.9–5.1)	
Median number of SNs on SPECT/CT, (IQR)	3.0 (3.0–4.0)			3.5 (3.0–4.8)		3.0 (2.3–3.8)	
*Intraoperative*							
Injection side fluorescein							
Left	7 (43.8)			4 (50.0)		3 (37.5)	
Right	9 (56.3)			4 (50.0)		5 (62.5)	
Median time injection fluorescein to first intraoperative imaging, minutes (IQR)	16 (15.0–21.5)			16.0 (14.5–24.3)		16.0 (16.0–21.5)	
Median OR time, minutes (IQR)	244 (222–256.5)			243 (223–255)		244 (213.3–255.5)	

Location fluorescein or ICG, *n* (% of patients)	Fluorescein	ICG		Fluorescein	ICG	Fluorescein	ICG
*Distribution of tracers in vivo*							
Cloquet’s	12 (75.0)	0 (0.0)		5 (62.5)	0 (0.0)	7 (87.5)	0 (0.0)
Obturator fossa	12 (75.0)	16 (100.0)		4 (50.0)	8 (100.0)	8 (100.0)	8 (100.0)
External iliac	10 (62.5)	10 (62.5)		4 (50.0)	6 (75.0)	6 (75.0)	4 (50.0)
Lateral to external iliac	11 (68.8)	2 (12.5)		5 (62.5)	2 (25.0)	6 (75.0)	0 (0.0)
Internal iliac	3 (18.8)	2 (12.5)		0 (0.0)	1 (12.5)	3 (37.5)	1 (12.5)
Common iliac	1 (6.3)	1 (6.3)		0 (0.0)	0 (0.0)	1 (12.5)	1 (12.5)
Medial to umbilical ligament / paravesical	1 (6.3)	4 (25.0)		0 (0.0)	3 (37.5)	1 (12.5)	1 (12.5)
Presacral / pararectal	0 (0.0)	4 (25.0)		0 (0.0)	2 (25.0)	0 (0.0)	2 (25.0)

Location to fluorescein injection side	Ipsilateral	Contralateral	Total	Total		Total	
*Histopathological (formalin fixed tissue)*							
Total LNs (incl SNs) removed, *n*	202	168	370	181		189	
Total tumor positive LNs, *n*	9	4	13	10		3	
Total LNs containing ICG, *n*	64	18	98	55		43	
Total LNs containing ICG *and* tumor-positive, *n*	9	2	11	9		2	
Total LNs containing fluorescein, *n*	10	0	10	5		5	

*ICG* indocyanine green, *IQR* interquartile range, *LN* lymph node, *MBq* Megabecquerel, *OR* operating room, *SN* sentinel node, *SPECT* single-photon emission computed tomography, *Tc* Technetium

### Intraoperative Imaging—Upper Leg Versus Abdominal Wall (Both Fluorescein)

Comparing upper leg and abdominal wall, the regions where fluorescein was observed were similar but showed some differences (left and right combined in Table 1; Fig. 3). The abdominal wall drainage was seen more frequently and in more regions than the upper leg. Both were seen mainly in Cloquet’s node (upper leg 63%, abdominal wall 88%) and lateral to external iliac (upper leg 50%, abdominal wall 75%). Although both visible, the abdominal wall pattern was seen in the obturator fossa in twice as many patients compared to upper leg (100% vs. 50%, respectively).

### Intraoperative Imaging—Lymph Fluid Leakage

In six patients (2/8 upper leg, 4/8 abdominal wall), fluorescein leakage was observed <1 cm of the cut lymph vessels. Leakage of lymph fluid >1 cm from the cut vessels was only identified when tracer was trapped between layers of fascia (*n* = 2) and after opening the bladder neck (*n* = 16; Supplementary 3). Leakage of ICG was not observed.

### Ex Vivo and Histopathological Evaluation

At the back table, in 9 of 167 (5%) pathological containers with freshly removed larger tissue samples, fluorescein was seen, and in 81 of 167 (49%) ICG. Two samples contained both fluorescein and ICG.

**Fig. 4:**
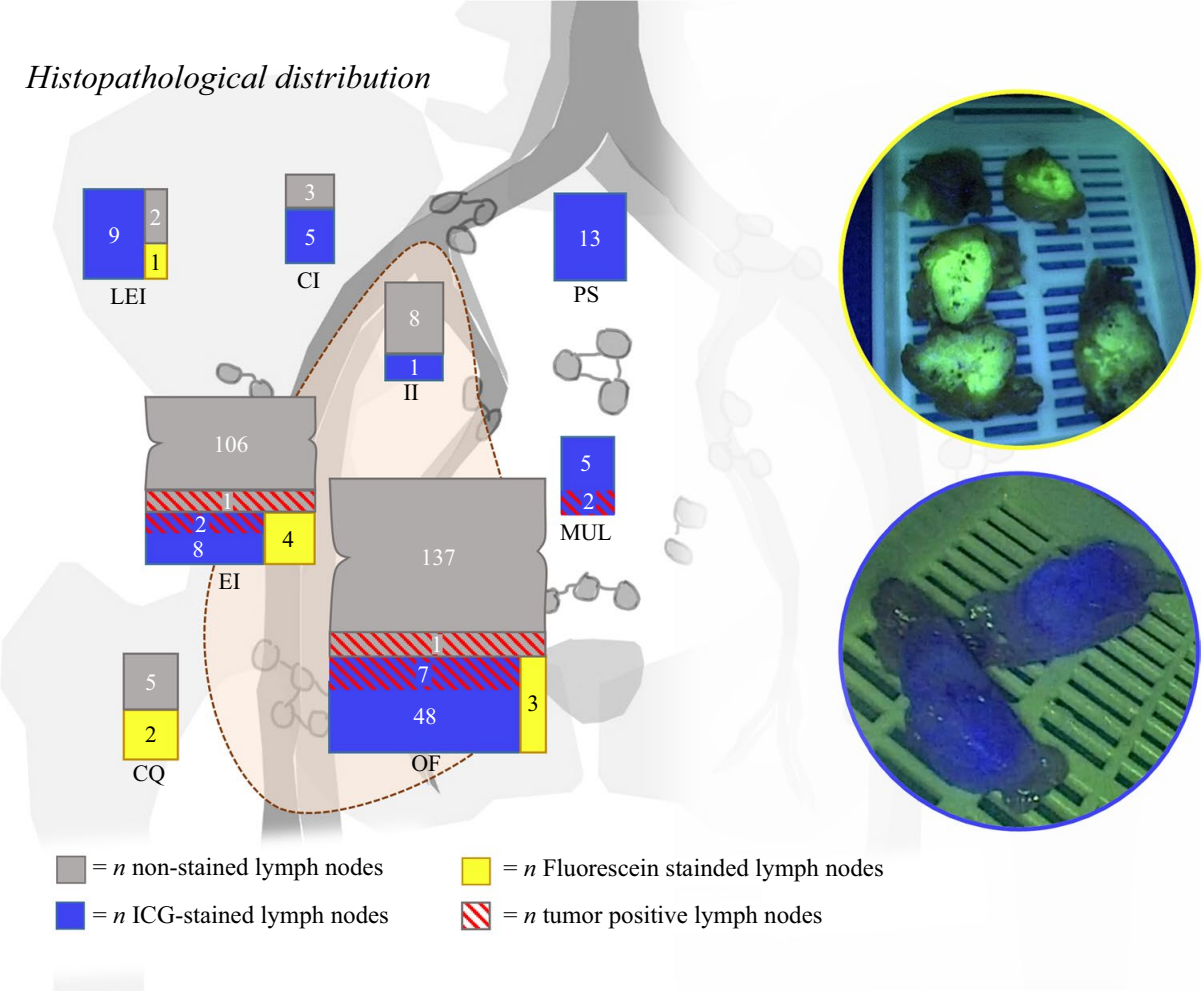
Unilateral representation of the distribution of detected lymph nodes with examples of KARL STORZ imaging filtered for ICG and fluorescein at histopathology. *CQ* Cloquet’s; *OF* obturator fossa; *EI* external iliac; *LEI* lateral to external iliac; *II* internal iliac; *CI* common iliac; *PS* presacral/pararectal; *MUL* medial to umbilical ligament/paravesical

Approximately 48 hours later, formalin-fixed nodal tissue samples were examined before paraffin-embedding for presence of fluorescein and ICG (*n* = 370). In 10 of 370 (3%), fluorescein was seen (all ipsilateral to injection side; total removed nodes ipsilaterally *n* = 202) (Table 1) and in 98 of 370 (26%) ICG (of which 64/98 (65%) ipsilateral to fluorescein injection), suggesting a fixation effect on the fluorescence signal. Although at the back table two samples contained both tracers, when examined at nodal level, none contained both fluorescein and ICG. Thirteen nodes were tumor-positive of which 11 (85%) were ICG stained (SNs) and 2 (15%) were not (Fig. 4). After paraffin-embedding, it was possible to identify ICG within LNs, but fluorescein staining was not specific (Supplementary 4).

### Safety and Follow-Up

The injection of fluorescein did not trigger any local or systemic reaction. Patients reported no postoperative discomfort or pain at the injection site, and no redness or swelling was seen during surgery, directly after surgery, or the following day. The presence of yellow skin discoloration lasted up to 2 days postoperatively (Supplementary 5).

Within 90 days postoperative, 12 of 16 (75%) patients suffered a complication grade Clavien-Dindo I, 5 of 16 (31%) Clavien Dindo II, and 2 of 16 (13%) Clavien-Dindo IIIa. Three serious adverse events (readmission) were observed in one patient (Table 2). All events were considered unrelated to fluorescein or ICG-^99m^Tc-nanocolloid administration.

**Table 2:** Complications according to Clavien–Dindo <90 days postsurgery

*Clavien Dindo I, n (% of 16 patients)*	
Lymphedema	12 (75%)
Paresthesia	2 (13%)
Mild allergic reaction to ciproxin	1 (6%)
*Clavien–Dindo II, n (%)*	
Lymphedema >2 months treated with edema therapy	1 (6%)
UTI treated with antibiotics	2 (13%)
TUC replacement	1 (6%)
Prolonged TUC in situ	1 (6%)
Infected urinoma (requiring readmission)	1 (6%)
Pulmonary embolism	1 (6%)
UTI with signs of bacteremia (requiring readmission)	1 (6%)
*Clavien–Dindo IIIa, n (%)*	
Cystoscopy before replacement of TUC	1 (6%)
Urinoma treated with nephrostomy catheter and abdominal drain (requiring readmission)	1 (6%)

*TUC* transurethral catheter; *UTI* urinary tract infection

One patient reported lymphedema in one leg (6%), seven in one or both legs together with the abdominal wall (44%), and four (25%) reported only lymphedema of the abdominal wall. Five men (35%) complained of a swollen scrotum—all independent of injection site and side. The lymphedema was reported to last between 2 days and 3 months. One patient was referred for specialized treatment regarding lymphedema of the abdominal wall. Postoperative PSA was undetectable in 13 of 16 (81%).

## Discussion

This prospective study demonstrated the feasibility of real-time, multispectral fluorescence imaging to visualize lymphatic drainage patterns from different anatomical locations within the ePLND-template. A first step in analyzing future applications where using multispectral imaging of drainage patterns will help distinguish patterns from healthy and diseased organs within the pelvis. Ultimately, the goal is to spare the lymphatics draining from the healthy organs and reduce morbidity. To translate fundamental multicolor imaging performed in mice, via porcine models, to humans, the clinically approved dyes ICG and fluorescein were used.^17,18^ Our in vivo findings indicate that lymphatic drainage patterns of prostate and other locations are in close relation within the ePLND-template.

When examining the distribution of the prostate compared with the upper leg or abdominal wall, it became apparent that some features from earlier observations in porcine models were lost.^18^ Lowering the fluorescein dose from 500 to 80 mg (for safety reasons) may have prevented intraoperative *nodal* visualization but did show fluorescein clearly in lymphatic vessels. Hence, the detection sensitivity for fluorescein was limited, and the distinct lymphatic separation observed in porcine models did not translate to the human situation. Still, the mapping of the drainage patterns was possible in regard to ePLND-template regions. That said, the observed patterns of skin or prostate injected tracers were clearly closely related in vivo. The main differences was that ICG was not observed around Cloquet’s, whereas fluorescein was absent in the presacral/pararectal regions (Fig. 3). This correlates with known literature where Cloquet’s node is regarded as the highest superficial node of the ilioinguinal basin when looking at lower limb drainage and is rarely reported to be a SN of the prostate (<1.1%).^24,25^

The in vivo distribution of leg or abdominal wall-injected fluorescein showed only ipsilateral staining, corroborating results in melanoma studies where contralateral nodes are only described in case reports where patients had prior surgery.^26–28^ The transient nature of fluorescein and high tissue attenuation of 515-nm fluorescent emissions made fluorescein detectability inferior compared with ICG for LN-imaging at the back table and after formalin fixation at histopathology. This may explain why only 10 of 202 of the ipsilateral nodes were clearly fluorescein-stained compared with the 64 ipsilateral ICG-stained nodes.

The lymphatic drainage pattern from the abdominal wall was similar to that from the upper leg, although the abdominal drainage pattern was seen more frequently and in more locations within the pelvis. This may be explained by a different drainage velocity or because the patterns slightly differ. The latter may explain why lymphedema of the abdominal wall was more frequently reported (75%) compared with lymphedema of the upper leg (50%).

In regards to complications, injection of 80 mg of fluorescein divided in two deposits in the skin did not result in redness, swelling, or other local or systemic events, confirming the results from Chang et al.^29^ Notably, our prevalence of lymphedema was higher than reported in the existing literature*.*^9^ The numbers may be underrated in current literature, because lymphedema is one of the least studied complications of prostate cancer treatment, despite its considerable impact on health-related quality of life*.*^9–12^ However, studies on gynecological cancers have reported high prevalence of lymphedema (up to 79%).^30,31^ Prostate cancer-related literature generally describes staff-reported lymphedema, which was lower compared with patient-reported.^32^ Data stem from predominantly retrospective studies. In this study, the lymphedema was patient-reported. Consequently, it should also be considered that patients’ perception of lymphedema was not verified by a physician, which may have caused bias. Other factors, such as body mass index, may play a role and are reported to have an increased risk of lymphedema in other cancers.^33,34^ In prostate cancer, the risk of complications in general is reported to be higher in overweight patients, but the association with lymphedema remains unknown.^35,36^

Limitations of the study include small sample size and that it was performed in a single center by different surgeons. In addition, determining whether the drainage patterns overlapped depended on various factors, including choosing the side with the most SNs on SPECT/CT and the location of the SN in vivo; all contribute to the already high interpatient variability of pelvic lymphatic anatomies.^37^ Furthermore, the fluorescence endoscope was maneuvered by the bedside assistant under verbal guidance, and in vivo fluorescence imaging deep in the pelvis could be challenging using this endoscope. The experimental multispectral imaging set-up described in this manuscript needs further optimization before it can be more widely applied. Hereby logistic refinement can be achieved by using a robot-integrated multispectral camera.^21^ Overall, precision imaging during surgery tends to cost extra operating room time but may ultimately help reduce costs later by minimizing the morbidity and reducing the recurrence rates.

Although the multispectral imaging was technically feasible, the in vivo closely related lymphatic drainage patterns and the fact fluorescein was relatively inferior indicate that sparing nondisease-related (fluorescein containing) lymphatic structures may result in undersampling cancer-containing LNs. Because overlapping patterns were not observed at nodal level at histopathology and lymphedema of the abdominal wall was seen more frequently, future studies should focus on possible tumor presence in nodes draining from the abdominal wall.

## Conclusions

Multispectral fluorescence imaging of two distinct drainage patterns was technically feasible during robot-assisted PCa surgery. Our initial in vivo findings indicate that within the ePLND-template, lymphatic drainage patterns of the prostate overlap with those of upper leg and abdominal wall. The properties of fluorescein render it unsuitable as a tracer for fluorescence histopathological confirmation.

This manuscript is also explained in video (Supplementary 6).

## Supplementary Information

Below is the link to the electronic supplementary material.

Supplementary file1 (DOCX 14 KB)

Supplementary file2 (DOCX 15 KB)

Supplementary file3 (DOCX 1960 KB)

Supplementary file4 (DOCX 1007 KB)

Supplementary file5 (DOCX 947 KB)

Supplementary file6 (MP4 156815 KB)
