# Correction: Multispectral Fluorescence Imaging as a Tool to Distinguish Pelvic Lymphatic Drainage Patterns During Robot-assisted Lymph Node Dissection in Prostate Cancer

**DOI:** 10.1245/s10434-024-16629-3

**Published:** 2024-12-13

**Authors:** Anne-Claire Berrens, Tessa Buckle, Matthias N. van Oosterom, Leon J. Slof, Pim J. van Leeuwen, Esther M. K. Wit, Hilda A. de Barros, Jakko A. Nieuwenhuijzen, Elise M. Bekers, Maarten L. Donswijk, Fijs W. B. van Leeuwen, Henk G. van der Poel

**Affiliations:** 1 Department of Urology, Netherlands Cancer Institute-Antoni van Leeuwenhoek Hospital, Amsterdam, The Netherlands; 2 Interventional Molecular Imaging Laboratory, Leiden University Medical Center, Leiden, The Netherlands; 3 Department of Urology, Amsterdam University Medical Center, VUmc, Amsterdam, The Netherlands; 4 Department of Pathology, Netherlands Cancer Institute-Antoni van Leeuwenhoek Hospital, Amsterdam, The Netherlands; 5 Department of Nuclear Medicine, Netherlands Cancer Institute-Antoni van Leeuwenhoek Hospital, Amsterdam, The Netherlands

**Correction to: Ann Surg Oncol** 10.1245/s10434-024-16423-1

The original online version of this article was corrected to improve the readibility of Table 1.

