# Supplementary material for: Multispectral Fluorescence Imaging as a Tool to Distinguish Pelvic Lymphatic Drainage Patterns During Robot-assisted Lymph Node Dissection in Prostate Cancer
Source: Ann Surg Oncol. 2024 Nov 19;32(2):1372–81. doi: 10.1245/s10434-024-16423-1 (PMC11698825; doi:10.1245/s10434-024-16423-1)
Supplement: Supplementary file 1 — Supplementary file1 (DOCX 14 KB) [file 10434_2024_16423_MOESM1_ESM.docx]

Supplementary 1. Detailed description of tracer injection

*Sentinel node (SN) identification*

Indocyanine Green(ICG)-99mTechnetium(Tc)-nanocolloid (based on Nanocoll® or Nanoscan®; GE Healthcare BV, Leiderdorp, the Netherlands;) was prepared by mixing 250 ICG in sterile water with 200 MBq ^99m^Tc-nanocolloid in 2 mL saline. The resulting formulation was administered approximately five hours prior to surgery in four deposits of 0.5 mL into the peripheral zone of the prostate using transrectal ultrasound guidance. At 15 min and two hours post tracer injection, lymphatic mapping was performed using static planar lymphoscintigraphy of the pelvic area, followed by single photon emission computed tomography (SPECT)/CT using a dual head gamma camera (Symbia T, Siemens, Erlangen, Germany). Planar images and SPECT, CT, and fused SPECT/CT including volume-rendering reconstructions were displayed using OsiriX medical imaging software (Pixmeo, Geneva, Switzerland). The images were reviewed by a nuclear medicine physician and the number and location of SNs were reported and communicated with the surgeon.

*Lymphangiography upper leg or abdominal wall*

In the operating room, after administering general anesthesia, fluorescein (80mg; 4ml of 2% fluorescein in saline 0.9%); ½-life 24 min) was injected unilaterally into the dermis in two deposits of 2ml each, either at the medial and lateral side of the upper leg (n=8) or at the left or right lower quadrant of the abdominal wall (n=8). The injection site was massaged to promote lymphatic flow. The side of injection (left/right) was chosen based on the side showing the most SNs on SPECT/CT to optimally investigate the possibility of imaging converging drainage patterns. On both sides ePLND and SN procedure was performed.

| **Fluorescent dye** | **λem max** | **Extinction coefficient** | **Fluorescence lifetime t^1/2^** | **Quantum yield** |
| --- | --- | --- | --- | --- |
| ICG | 820 nm | 1.8 x 10^5^ (HSA) | 0.97 (DMSO) | 5 (HSA) |
| Fluorescein | 515 nm | 0.4 x 10^5^ (HSA) | 4 (H2O) | 12 (HSA) |

*from Meershoek et al. 2020 [20]
