# Supplementary material for: Multispectral Fluorescence Imaging as a Tool to Distinguish Pelvic Lymphatic Drainage Patterns During Robot-assisted Lymph Node Dissection in Prostate Cancer
Source: Ann Surg Oncol. 2024 Nov 19;32(2):1372–81. doi: 10.1245/s10434-024-16423-1 (PMC11698825; doi:10.1245/s10434-024-16423-1)
Supplement: Supplementary file 2 — Supplementary file2 (DOCX 15 KB) [file 10434_2024_16423_MOESM2_ESM.docx]

Supplementary 2. Patient demographics

| ***Upper leg*** | **Age** | **Clinical  TNM stage** | **MRI  T stage** | **iPSA, ng/mL** | **Biopsy  ISUP grade group** | **Risk of LNI Briganti 2012** | **Pathological  TNM stage** | **Pathological  ISUP  grade group** | **Resection  margin** | **PSA at first follow up visit, ng/mL** |
| --- | --- | --- | --- | --- | --- | --- | --- | --- | --- | --- |
| Patient 1 | 66 | cT2N0M0 | T2b | 7.8 | 3 | 9.8 | pT2N0 | 3 | R0 | <0.01 |
| Patient 2 | 68 | cT2N0M0 | T3a | 22.8 | 3 | 21.9 | pT3aN1 | 2 | R1 | 1.32 |
| Patient 3 | 69 | cT2bN0M0 | T2 | 13.0 | 4 | 17.0 | pT2cN1 | 3 | R0 | <0.01 |
| Patient 4 | 71 | cT2N0M0 | T3a | 42.0 | 3 | 38.0 | pT3aN1 | 4 | R1 | 0.18 |
| Patient 5 | 68 | cT2N0M0 | T3b | 16.0 | 3 | 32.7 | pT3bN0 | 3 | R1 | <0.01 |
| Patient 6 | 75 | cT1cN0M0 | T2 | 7.7 | 3 | 12.4 | pT2N0 | 3 | R0 | 0.5 |
| Patient 7 | 57 | cT1cN0M0 | T2 | 16.8 | 5 | 9.4 | pT3aN0 | 5 | R0 | 0.07 |
| Patient 8 | 61 | cT2N1M0 | T3b | 14.6 | 2 | 12.6 | pT3bN1 | 2 | R1 | <0.01 |
| ***Abdominal wall*** | | | | | | | | | | |
| Patient 9 | 60 | cT3aN0M0 | T3b | 24.5 | 2 | 50.7 | pT3bN0 | 3 | R1 | <0.01 |
| Patient 10 | 64 | cT2N0M0 | T3a | 5.9 | 3 | 15.6 | pT3aN0 | 2 | R0 | <0.05 |
| Patient 11 | 67 | cT2N0M0 | T2 | 14.0 | 5 | 19.0 | pT2N1 | 3 | R0 | <0.006 |
| Patient 12 | 68 | cT2N0M0 | T3a | 5.52 | 2 | 7.2 | pT3aN0 | 2 | R0 | <0.01 |
| Patient 13 | 62 | cT2N0M0 | T2 | 17.5 | 2 | 18.8 | pT3aN0 | 3 | R0 | <0.01 |
| Patient 14 | 53 | cT1cN0M0 | T3a | 45.6 | 5 | 40.3 | pT2N0 | 3 | R0 | <0.01 |
| Patient 15 | 65 | cT2N0M0 | T3a | 19.3 | 3 | 34.0 | pT3bN1 | 3 | R0 | <0.01 |
| Patient 16 | 71 | cT2N0M0 | T3a | 7.9 | 5 | 28.5 | pT2N0 | 2 | R0 | <0.1 |
| *iPSA = initial prostate-specific antigen; ISUP = International Society of Urological Pathology; LNI = Lymph node involvement* | | | | | | | | | | |
