# Supplementary material for: Multispectral Fluorescence Imaging as a Tool to Distinguish Pelvic Lymphatic Drainage Patterns During Robot-assisted Lymph Node Dissection in Prostate Cancer
Source: Ann Surg Oncol. 2024 Nov 19;32(2):1372–81. doi: 10.1245/s10434-024-16423-1 (PMC11698825; doi:10.1245/s10434-024-16423-1)

Supplementary 3. A) Before, during and after fluorescein stained lymph vessels connecting Cloquet’s with the obturator fossa are being severed (fluorescein imaging). B) Lymph fluid leakage when trapped between fascia in white light, Firefly integrated camera imaging and fluorescein imaging.


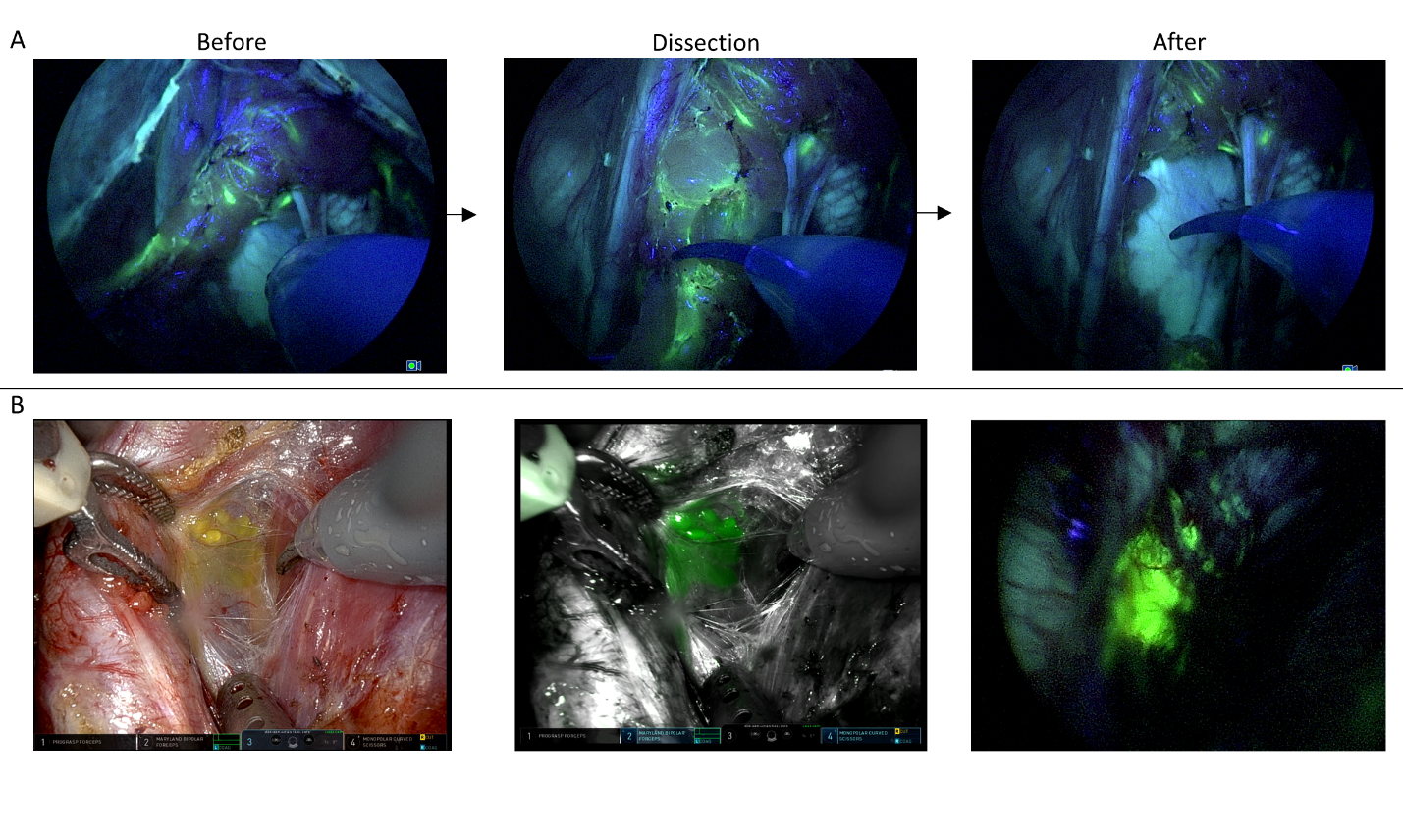

Supplement: Supplementary file 3 — Supplementary file3 (DOCX 1960 KB) [file 10434_2024_16423_MOESM3_ESM.docx]
