# Supplementary material for: Multispectral Fluorescence Imaging as a Tool to Distinguish Pelvic Lymphatic Drainage Patterns During Robot-assisted Lymph Node Dissection in Prostate Cancer
Source: Ann Surg Oncol. 2024 Nov 19;32(2):1372–81. doi: 10.1245/s10434-024-16423-1 (PMC11698825; doi:10.1245/s10434-024-16423-1)

Supplementary 4. A) Fluorescein imaging after paraffin embedding in a Fluorescein stained node at histopathology. B) Fluorescein imaging after paraffin embedding in a control patient


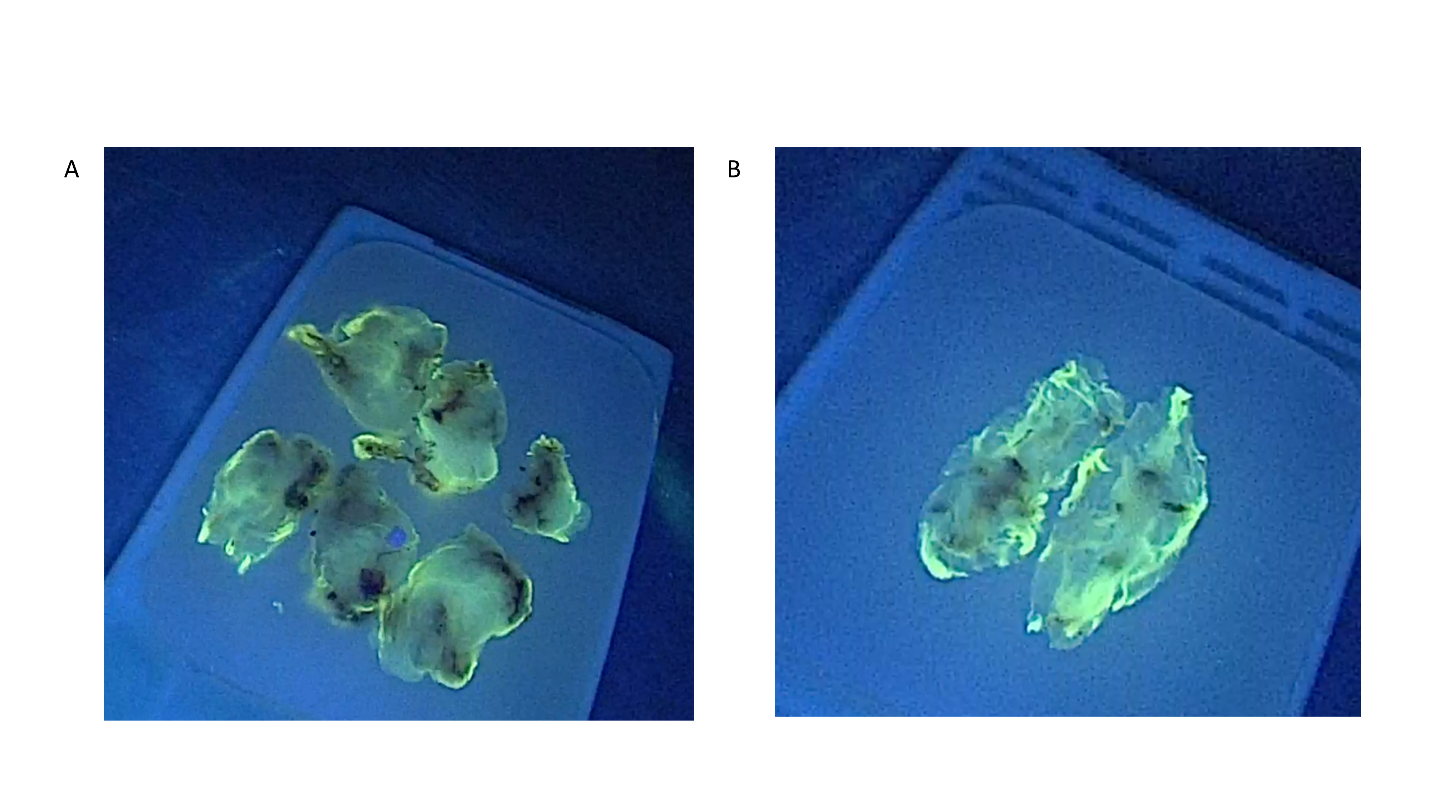

Supplement: Supplementary file 4 — Supplementary file4 (DOCX 1007 KB) [file 10434_2024_16423_MOESM4_ESM.docx]
