# Supplementary material for: Multispectral Fluorescence Imaging as a Tool to Distinguish Pelvic Lymphatic Drainage Patterns During Robot-assisted Lymph Node Dissection in Prostate Cancer
Source: Ann Surg Oncol. 2024 Nov 19;32(2):1372–81. doi: 10.1245/s10434-024-16423-1 (PMC11698825; doi:10.1245/s10434-024-16423-1)

Supplementary 5. White light and UV-light imaging of the injection site directly postoperative and 1 day post-surgery.


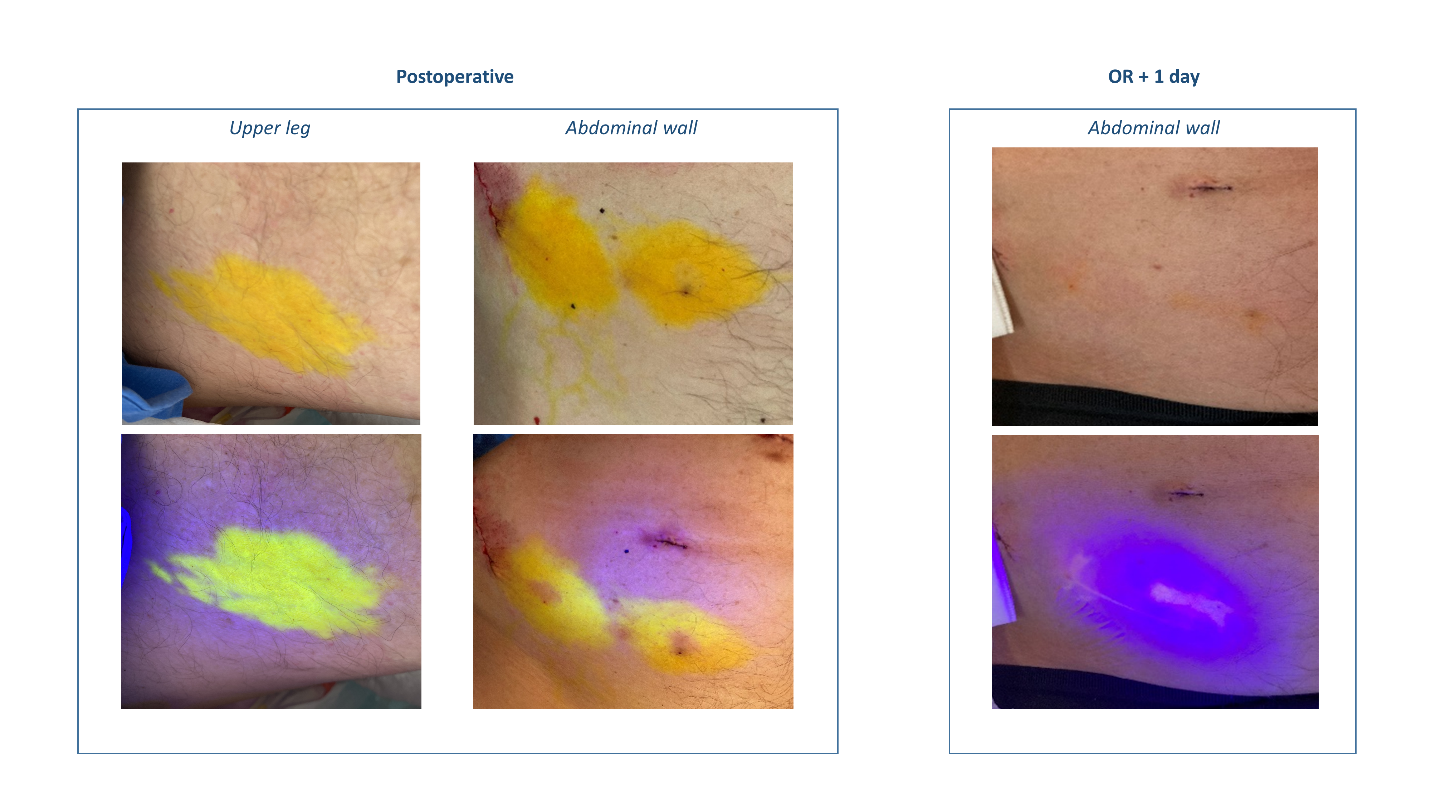

Supplement: Supplementary file 5 — Supplementary file5 (DOCX 947 KB) [file 10434_2024_16423_MOESM5_ESM.docx]
